# Microfluidic chips in female reproduction: a systematic review of status, advances, and challenges

**DOI:** 10.7150/thno.97301

**Published:** 2024-07-15

**Authors:** Tong Wu, Jinfeng Yan, Kebing Nie, Ying Chen, Yangyang Wu, Shixuan Wang, Jinjin Zhang

**Affiliations:** 1 Department of Obstetrics and Gynecology, National Clinical Research Center for Obstetrics and Gynecology, Tongji Hospital, Tongji Medical College, Huazhong University of Science and Technology, Wuhan, China.; 2 Key Laboratory of Cancer Invasion and Metastasis (Ministry of Education), Hubei Key Laboratory of Tumor Invasion and Metastasis, Tongji Hospital, Tongji Medical College, Huazhong University of Science and Technology, Wuhan, China.; 3 School of Materials Science and Engineering, Huazhong University of Science and Technology, Wuhan, China.; 4 College of Animal Science and Technology, Sichuan Agricultural University, Sichuan, China.

**Keywords:** female reproduction, microfluidic chip, ovary, engineering, fertility

## Abstract

The female reproductive system is essential to women's health, human reproduction and societal well-being. However, the clinical translation of traditional research models is restricted due to the uncertain effects and low efficiency. Emerging evidence shows that microfluidic chips provide valuable platforms for studying the female reproductive system, while no paper has ever comprehensively discussed the topic. Here, a total of 161 studies out of 14,669 records are identified in PubMed, Scopus, Web of Science, ScienceDirect and IEEE Xplore databases. Among these, 61 studies focus on oocytes, which further involves culture, cell surgeries (oocyte separation, rotation, enucleation, and denudation), evaluation and cryopreservation. Forty studies investigate embryo manipulation via microfluidic chips, covering *in vitro* fertilization, cryopreservation and functional evaluation. Forty-six studies reconstitute both the physiological and pathological statuses of *in vivo* organs, mostly involved in placenta and fetal membrane research. Fourteen studies perform drug screening and toxicity testing. In this review, we summarize the current application of microfluidic chips in studying the female reproductive system, the advancements in materials and methods, and discuss the future challenges. The present evidence suggests that microfluidic chips-assisted reproductive system reconstruction is promising and more studies are urgently needed.

## Introduction

**Figure 1:**
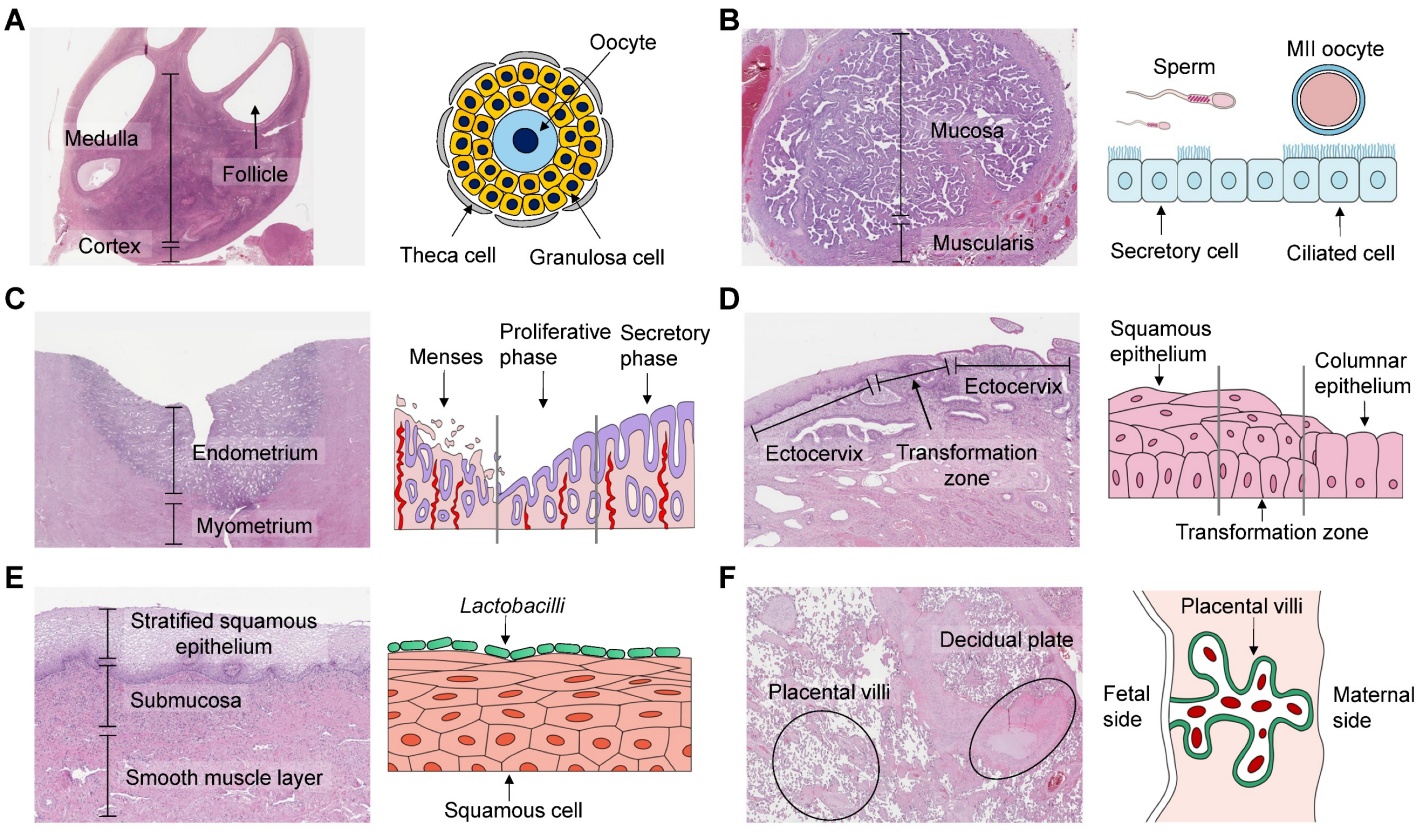
Histological images and schematics of human female reproductive organs. (A) The ovary comprises a central medulla and peripheral cortex. Oocytes are surrounded by granulosa cells and theca cells inside follicles, and their interactions are essential for hormonal secretion and gamete maturation. (B) The ampulla of the fallopian tube has many branching folds, and provides a site for fertilization. Secretory cells produce nutrients, while ciliated cells transport the fertilized egg towards the uterus. (C) The uterus plays a pivotal role in implantation and conceptus development. The uterine endometrium undergoes cyclic growth and regression under the control of ovarian hormones, including the menstrual, proliferative, and secretory phases. (D) The cervix can be divided into the endocervix, transformation zone, and ectocervix. It prevents microorganisms from entering, screens sperm, and lets menstrual blood flow out. (E) The vagina is covered by multiple squamous cell layers that allow bacterial colonization. Lactobacilli are the predominant vaginal microbiota. (F) The placenta-decidual interface mediates metabolic exchange between the developing fetus and the mother. In humans, the placenta consists of the trophoblastic epithelium covering the villi, the chorionic connective tissue and the capillary endothelium, which are all of fetal origin. The human histological images are reproduced with permission from the Human Protein Atlas, copyright 2023 (https://www.proteinatlas.org/humanproteome/tissue).

The female reproductive system (FRS), mainly comprising the ovary, fallopian tube, uterus, and vagina, plays a crucial role in affecting menstrual cycle, fertility, and pregnancy. The unique morphology and structure of these organs ensure their ability to fulfill individual functions and maintain reciprocal communications (Figure 1). For instance, the ovary synthesizes and secretes steroid hormones that regulate the uterine endometrium 1. The fallopian tube is responsible for collecting ovulated matured oocytes and transporting zygotes to the uterine cavity. These years, much efforts have been made to advance the knowledge of the FRS, particularly pertaining to oocytes, embryos, and fetal-maternal interfaces. However, translational research to clinical practice faces challenges stemming from structural differences, deficient design, and inadequate reporting 2. Of significant concern are the research modalities, like animal models, and two-dimensional (2D) *in vitro* culture approaches 3. Animal experiments may cause pain, and require substantial labor. The interspecies differences and complexity of certain organs, such as the highly diverse placenta, make it unsuitable to employ animal models 4, 5. As for 2D culture, its condition is significantly different from the *in vivo* microenvironment, thus distorting cellular morphology and behavior. Therefore, it is imperative to develop more efficient experimental methods to enhance translational research and promote women's health 6.

**Figure 2:**
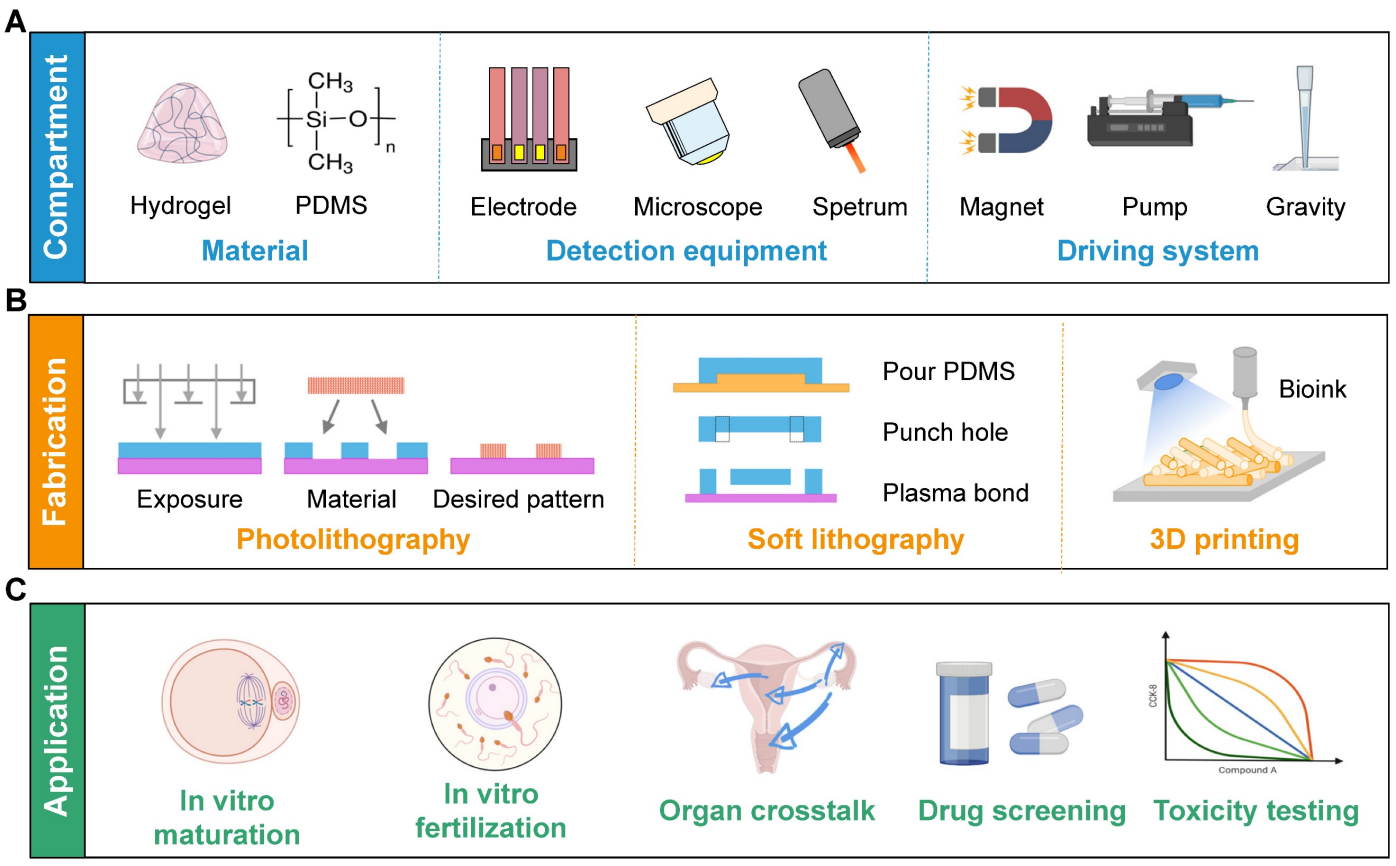
Diagrams of the compartments, fabrication methods, and applications of microfluidic chips in the FRS. (A) Hydrogel and PDMS are frequently used materials. The functions can be extended by various detection equipment. Continuous flow is achieved by a magnetic field, peristaltic pump, or gravity. (B) Commonly-used manufacturing processes include photolithography, soft lithography, and 3D printing. (C) The main applications focus on assisted reproduction techniques, organ crosstalk, drug screening and toxicity testing. Abbreviations: 3D: three-dimensional; FRS: female reproductive system; PDMS: polydimethylsiloxane.

With the advancement of biotechnology, the microfluidic chip, also known as organ-on-a-chip, has proven to be more instrumental than traditional experimental approaches in regenerative medicine, drug screening, and toxicity testing. It effectively mimics the *in vivo* microenvironment by incorporating dynamic fluid flow, thus enabling the long-term survival of cells. Furthermore, it can be coupled with microscopes, electrode arrays, or sensors to support real-time imaging, high-throughput analysis, and biochemical testing, respectively (Figure 2) 7. Nevertheless, the distinct characteristics of the FRS set it apart from other organs. First, oocytes and follicles are considerably larger than ordinary cells, and they gradually increase in size 8. Second, the continuous developmental process from follicles to embryos is complicated to achieve. Third, the female reproductive tract undergoes dramatic morphological and biochemical changes in different estrus stages. Consequently, utilizing the microfluidics technique in the FRS holds great importance but remains a challenge.

In this review, we systematically searched five databases using a set of selected keywords based on the Preferred Reporting Items for Systematic Reviews and Meta-Analyses guidelines. We offer a comprehensive overview of the microfluidic chip's applications in the FRS, summarizing the functions, experimental designs, fabrication methods, and main findings. We further presented prospects and challenges for advancing microfluidic chips in this domain.

## Methods

### Data sources

This systematic review followed the Preferred Reporting Items for Systematic Reviews and Meta-Analyses protocol 9, and was registered under the Open Science Frame REGISTRIES (ID: osf.io/a4dkn). A comprehensive search was systematically executed across PubMed, Scopus, Web of Science, ScienceDirect, and IEEE Xplore databases, with publication years spanning from January 2010 to December 2023. The search was not limited by language.

### Retrieval strategy

The search terms were formulated based on the widely recognized PICO (Population, Intervention, Comparison, Outcome) principle: (P) all models relevant to the FRS; (I) the microfluidic chip or organ-on-a-chip; (O) viability and function of the female reproductive tract cells, as well as the fabrication information. Eventually, we employed the following retrieval formula: (“ovary” OR “follicle” OR “oocyte” OR “fallopian tube” OR “oviduct” OR “uterus” OR “endometrium” OR “womb” OR “hystera” OR “vagina” OR “cervix” OR “embryo” OR “placenta” OR “fetal membrane” OR “amnion” OR “decidua”) AND (“microfluidic” OR “chip”) (Table S1).

### Manually searching for references

While the initial database search is systematic and covers a wide range of sources, it is possible that some relevant studies might not be covered due to variations in indexing, keyword usage, or database scope. Therefore, additional papers were identified by manually looking through the reference lists of identified studies, as well as reviewing related articles that might not have been initially identified 10. This approach ensures that no relevant research is overlooked, and that the systematic review is as comprehensive as possible.

### Eligibility criteria

Documents were included based on the criteria: (i) accessible, peer-reviewed papers published between January 2010 and December 2023; (ii) use of microfluidic platforms or organ-on-a-chip for female reproductive studies; (iii) use of mammalian cells or tissues. Studies were excluded for the following reasons: (i) review papers, conference papers, commentaries, and communications; (ii) non-mammals; (iii) tumor research; (iv) *ex vivo* models using perfusion systems without chips; (v) mere computational models without experimental validation; (vi) irrelevant to the female reproduction tract. Two authors (T.W. and J.F.Y.) independently selected studies according to the above criteria. The titles and abstracts were manually examined, and the reasons for excluding or including the articles were recorded. Discrepancies between the two authors were discussed with a senior author (S.X.W.), who made the final decision.

### Data extraction

Basic information included full titles, first authors, publication years, affiliations, countries, and keywords. Outcomes of interest included the animal models, fabrication methods, used biomaterials, chip designs, types of cells, key groups, and main findings. The above items were carefully reviewed by J.J.Z. and S.X.W.

### Visualization and bibliometric analysis

We used the VOSviewer to analyze research papers in medicine, geology, and ecology 11. All keywords (titles, abstracts, and authors' keywords) of eligible studies from the Web of Science database were used to analyze the co-occurrence. Keywords with the same meaning, such as “chip”, “microfluidic chip”, and “microfluidics” were combined into a single frequency. The word cloud was visualized by the OmicShare online tool.

## Results

**Figure 3:**
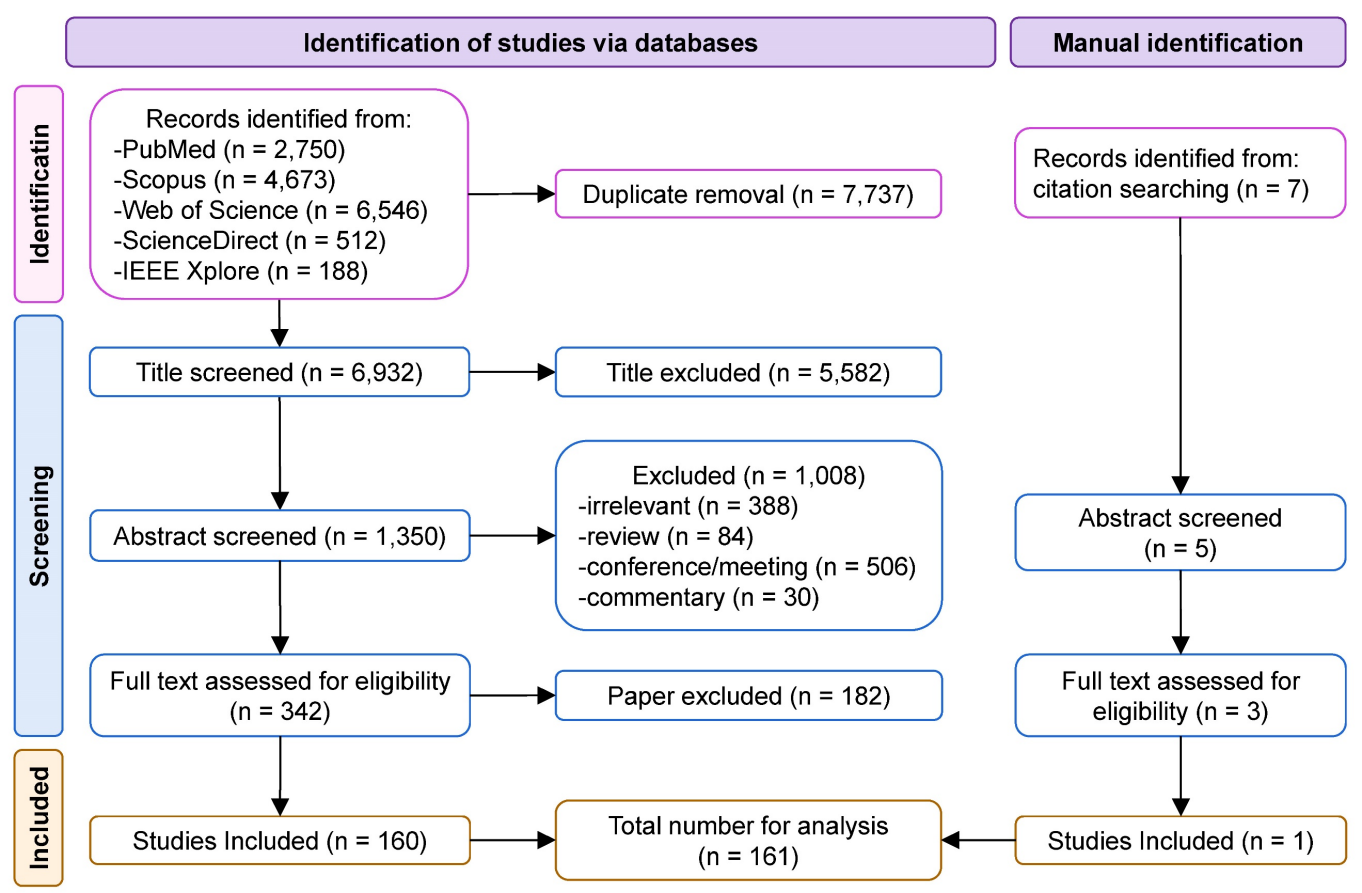
Flow diagram of the systematic article selection.

A flowchart of the search procedure is shown in Figure 3. The initial electronic database search resulted in 14,669 papers, of which 6,932 remained after removing duplicates. We excluded 5,582 and 1,008 papers after screening the title and abstract, respectively. The text of 342 studies was carefully assessed, and 182 studies that did not meet the inclusion criteria were excluded. An additional study was retrieved by manually searching the references. Finally, 161 papers were included as eligible for further analysis.

### Characteristics of search results

**Figure 4:**
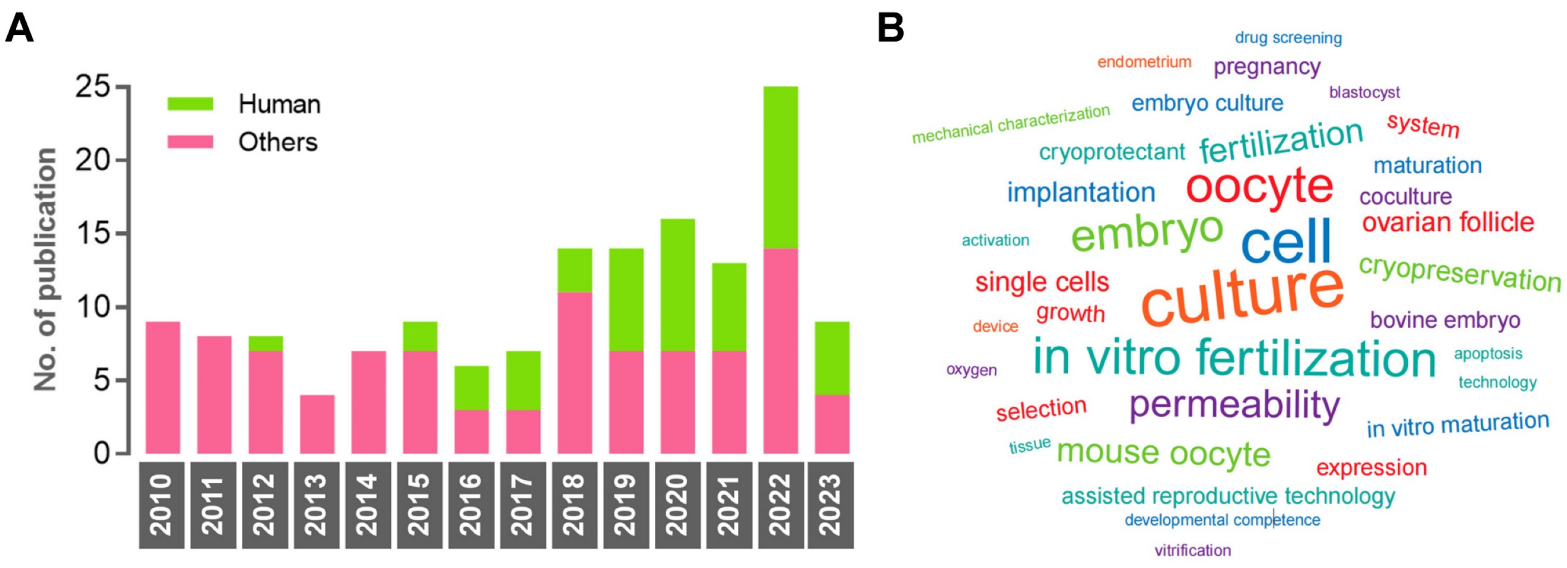
Characteristics of the eligible studies. (A) Histogram of publication numbers showing the use of human samples between 2010 and 2023. (B) The research diversity is reflected by a word cloud. A larger font means the word is more frequently used.

The number of documents showed an increasing trend between 2010 and 2023, while the percentage of studies using human samples fluctuated between 12.5% and 57.1% (Figure 4A). The numbers of non-human samples were as follows: mouse (n = 61), bovine (n = 19), and porcine (n = 15). The USA emerged as the leading country in contributing to this research area (n = 47, 30.3%), followed by China (n = 45, 29.0%) and Japan (n = 20, 12.9%). In terms of institutions, Nagoya University published the highest number of original articles (n = 13). The word cloud map revealed that the most frequently mentioned keywords included culture, cell, oocyte, *in vitro* fertilization, embryo, mouse oocyte, and permeability (Figure 4B).

### *In vitro* culture and maturation of oocytes

**Figure 5:**
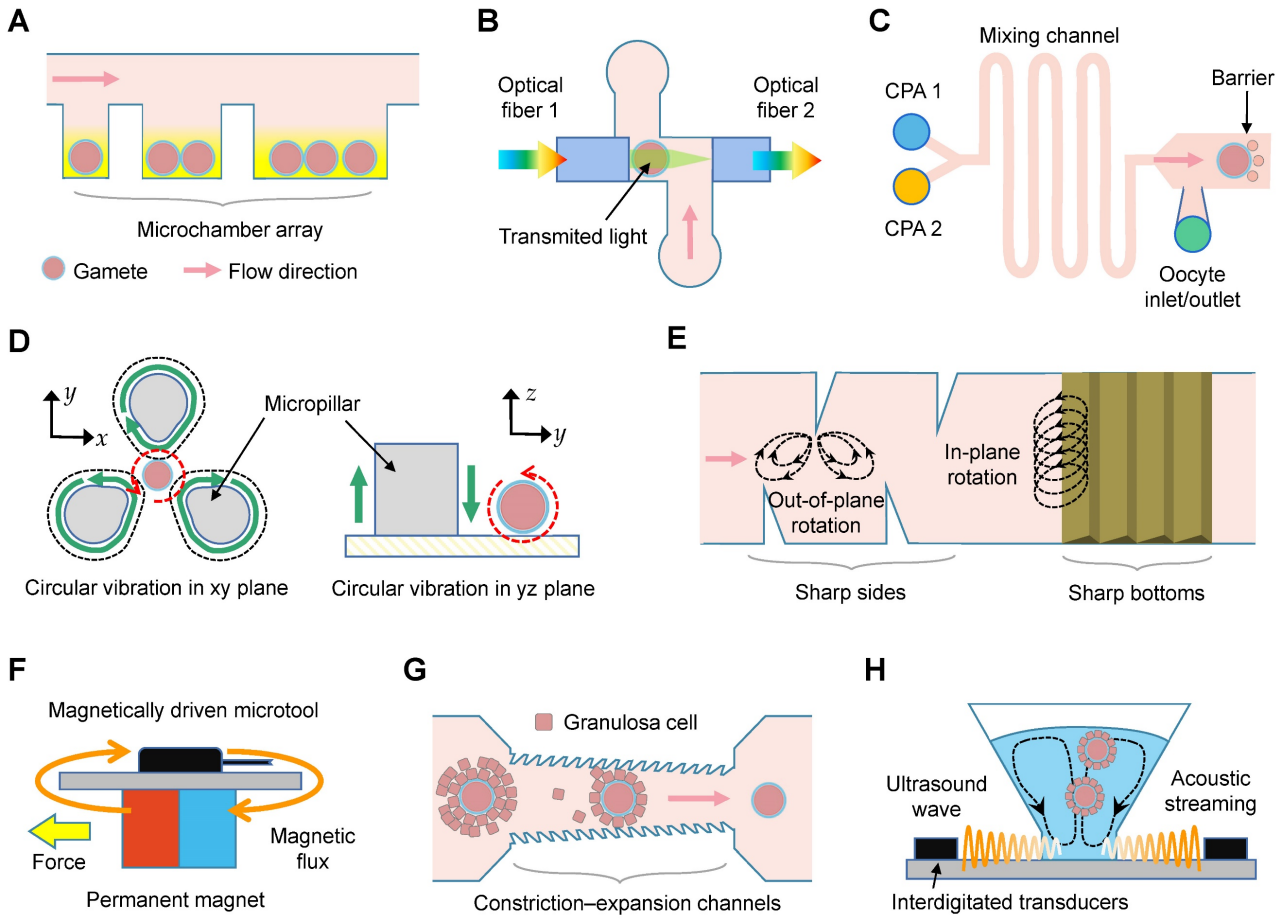
Culture, observation, and manipulation of oocytes using microfluidics. (A) Oocytes are trapped in an array of microchambers for *in vitro* maturation. (B) Schematic of the microfluidics for spectrophotometric characterization. (C) Schematic showing the linear cryoprotectant (CPA) addition method using the microfluidics. (D) Oocyte rotation based on vibration-induced flow: (left) vertical plane rotation and (right) focal plane rotation. (E) Streaming is induced by the oscillations of sharp sides and bottoms. (F) Horizontal polar drive using magnetically driven microtools to precisely control oocytes. (G) Schematic of the constriction-expansion channels for oocyte denudation. The height of inlets is taller than the outlets, and constrictions are featured by jagged surface. (H) Streaming inside the microwells is induced by the ultrasound wave, which is produced by digital transducers, to shake and squeeze cells.

The microfluidics used for oocyte culture and maturation were reported in nine studies (Table 1), employing denuded oocytes (n = 3) 12-14, cumulus-oocyte complexes (n = 3) 15-17, ovarian tissues (n = 2) 18, 19, and preantral follicles (n = 1) 20. Among these, one study explored the impact of single and group settings on maturation by trapping varying numbers of oocytes (Figure 5A) 12. Six studies conducted a comparative analysis of viability, diameter, and hormone levels between oocytes matured in microfluidic chips and those matured in traditional 2D dish or static culture systems. The effects of electric fields 13, flow rates 17-19, and collagen/alginate combinations 20 on oocyte growth were also examined.

### Oocyte evaluation

Twenty-three studies aimed to evaluate the properties of oocytes (Table 2). Eight studies deformed oocytes to test their responses to mechanical stimuli 21-28. Notably, only one study specially applied injection force 21, whereas the remaining seven studies used compression or suction force. Furthermore, six studies investigated cellular membrane permeability 29-34. Three studies examined the structural characteristics 35-37, and the optical spectrum of oocytes (n = 3; Figure 5B) 38-40. Less frequently reported properties were electrical impedance (n = 2) 41, 42, and oxygen concentration (n = 1) 43.

### Cryopreservation of oocytes

Eight studies used microfluidic chips to minimize osmotic and toxic damages during cryopreservation (Table 3) 44-51. Four studies employed a manual step-wise protocol to manage oocyte cryopreservation 44, 46, 47, 51. Five studies compared different methods for loading and removing cryoprotectants (CPAs) 44, 45, 48-50, and found that concave loading and convex unloading of CPAs produced the most favorable results (Figure 5C) 50.

### Cell surgeries

Twenty-one studies examined techniques for oocyte separation (n = 4; Table 4) 52-55, rotation (n = 8) 56-63, enucleation (n = 7) 64-70, and denudation (n = 2) 71, 72. Three types of microfluidic devices were designed to isolate single oocyte based on optical sensor feedback 52, 68, sedimentation rate 54, or oocyte size 55. Three studies successfully achieved precise oocyte orientation through permanent magnets and voltage-generated flow streaming 56, 58, 62, while microstructures within the chips were utilized in another four studies for the same purpose (Figure 5D-E) 59-61, 63. Of the studies focusing on oocyte enucleation, two used electric field-induced driving forces 64, 70, and four used magnetically-driven microtools (Figure 5F) 65, 66, 68, 69. Microfluidic devices to denudate oocytes within cumulus cells were included in two studies, using either microchannel constriction (Figure 5G) 71, or an acoustic radiation force (Figure 5H) 72.

### *In vitro* fertilization and culture of primary embryos

**Figure 6:**
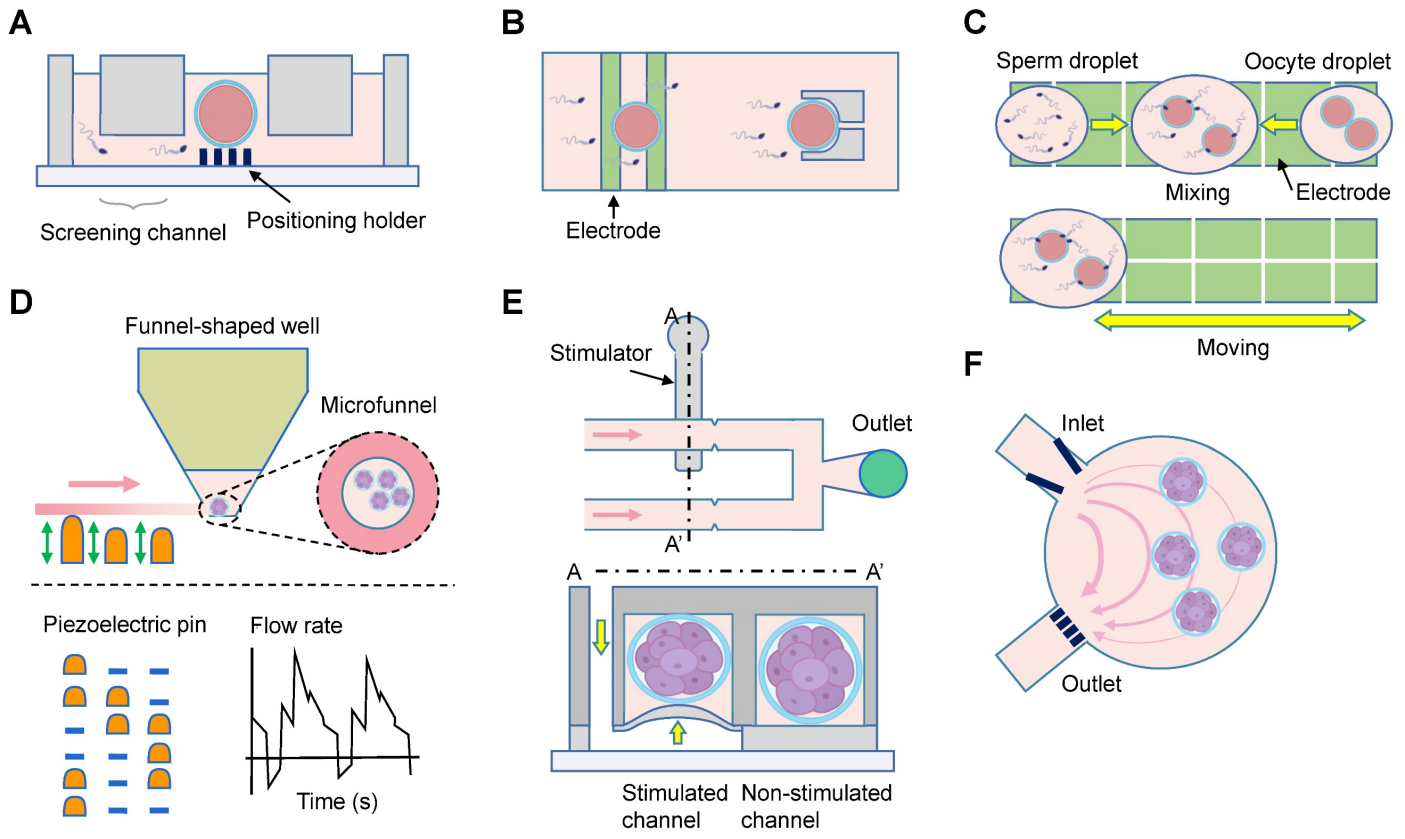
Investigations on *in vitro* fertilization and embryo culture. (A) The motile sperm swim through the motility screening channels spontaneously to reach the central oocytes. (B) The oocytes and sperm are first trapped on the electrodes for about 1 minute. Afterwards they are co-incubated for 1 hour in the micro-structures for natural insemination. (C) Droplet manipulation on a digital microfluidic platform. The sperm and oocyte droplets approach each other, and then mix for* in vitro* fertilization. The embryos are dynamically cultured with an automatic program. (D) The embryos are cultured in microfunnels, and the flow is generated by the actuation sequence of piezoelectric pins. (E) The microfluidic device comprises a control module, a stimulated microchannel, and a non-stimulated microchannel. Pressure is applied to the stimulated channel by a syringe pump. (F) Embryos are guided by the V-shaped structure, and trapped in a circular chamber with the help of grids. The flow profile under constant pumping is shown.

Embryo culture is one of the most important steps during *in vitro* fertilization. The included 25 studies could be categorized into mono- and co-culture systems (Table 5) 73-97. Among the 18 mono-culture studies, 13 compared the microfluidic system with Petri dish approaches, with nine studies reporting beneficial effects of the microfluidics 74, 76, 79-81, 84-86, 89. Four studies integrated oocyte capture, sperm selection, *in vitro* fertilization, and embryo culture functions using specific chamber design (Figure 6A) 75, 78, or dielectrophoretic approach (Figure 6B-C) 81, 88. Heo *et al.* provided embryos with fluid mechanical stimulation and the media could be easily loaded and unloaded by simple pipetting (Figure 6D) 76. One study used Pluronic F127 to conduct surface functionalization and block non-selective adsorption of culture medium, with comparable blastocyst and implantation rates to those in Petri dish 89. The pre- and post-implantation development has also been achieved in a confined microfluidic environment (Figure 6F) 79. Improved outcomes of embryonic development were demonstrated in all the seven co-culture systems. Oviductal epithelial cells were employed in four studies 92, 94, 95, 97, while uterine endometrial stromal cells were utilized in two studies 91, 93, and mesenchymal stem cell in one study 96.

### Embryo evaluation and cryopreservation

Eleven and four studies were conducted to characterize embryo 98-108, and cryopreserve embryos 109-112, respectively (Table 2-3). The evaluations included morphological changes (n = 3) 98, 100, 101, viability (n = 2) 102, 103, and mechanics (n = 1; Figure 6E) 99. Two groups investigated the oxygen consumption of embryos 104, 105. Furthermore, the secretion of human chorionic gonadotropin beta, inflammatory factors, and glucose was studied at a single-embryo level, demonstrating in-depth metabolic and hormonal analysis of the embryo development 106-108. Lastly, four studies compared embryo cryopreservation using microfluidic chips and manual operation, with similar outcomes 109-112.

### Simulation of *in vivo* organs

**Figure 7:**
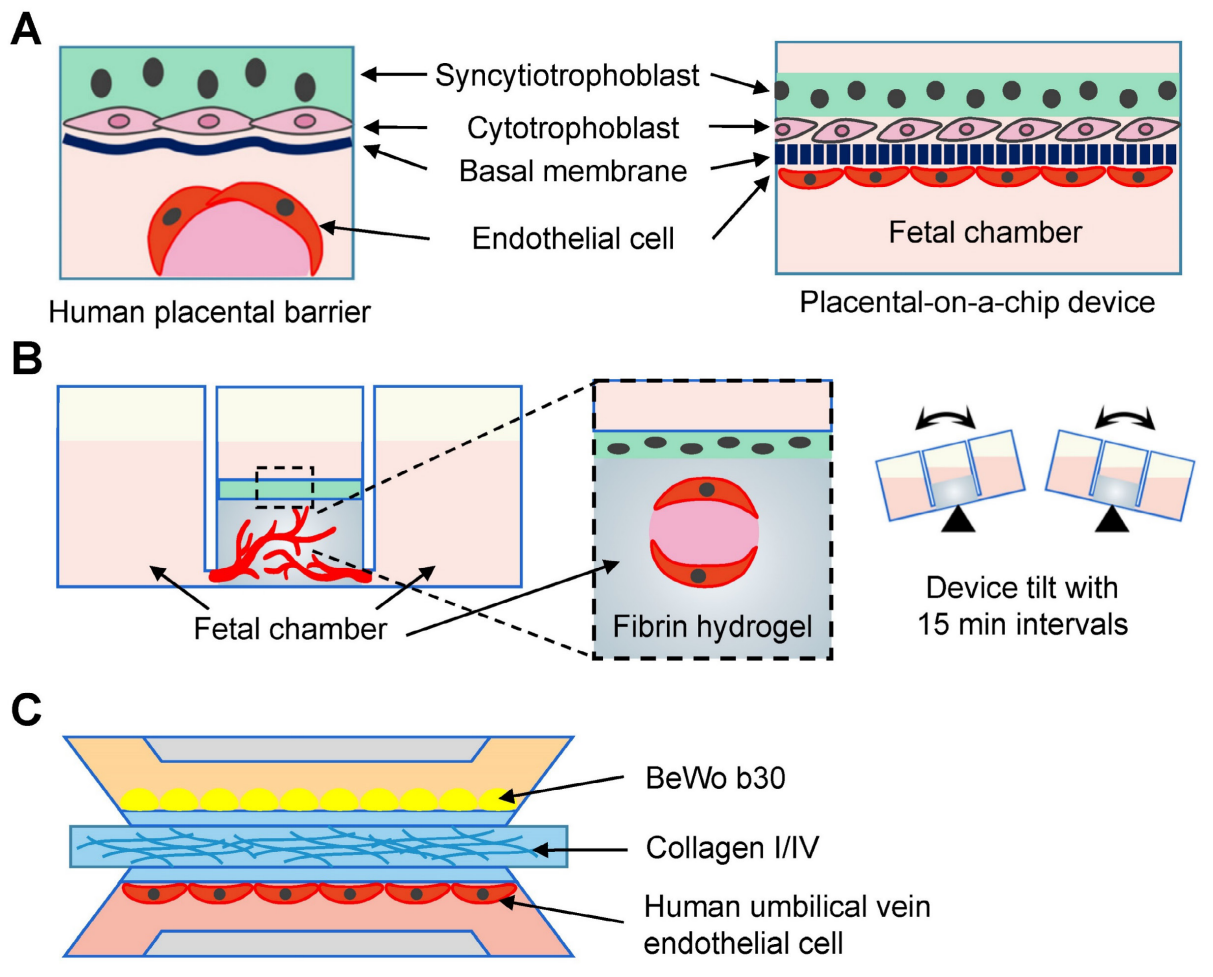
Reconstruction of placental model using the microfluidic chips. (A) Endothelial cells and trophoblasts are co-cultured in close apposition on the opposite sides of the membrane to form the placenta-on-a-chip device. (B) The term placenta is modeled using an IFlowPlate co-culture model, which consists of a differentiated syncytiotrophoblast monolayer cultured on a fibrin hydrogel. The endothelial cells inside the gel self-assemble to form perfusable vasculature. (C) A placental microfluidic chip in the OrganoPlate 3-lane.

**Figure 8:**
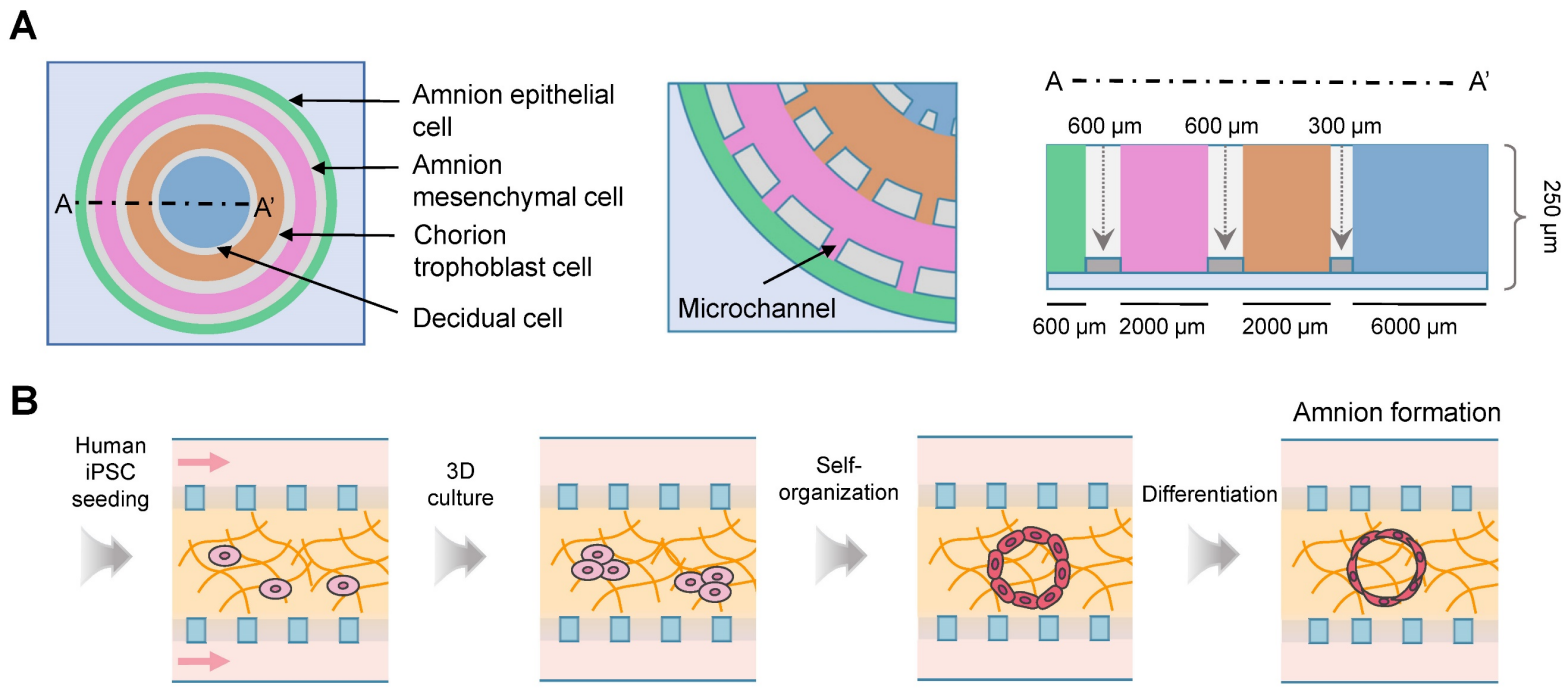
Recreation of fetal membrane-decidual or choriodecidual interface using the microfluidics. (A) The fetal membrane interface organ-on-chip is composed of four circular chambers connected by microchannels. The width of each chamber replicates the natural thickness of the fetal membrane. (B) Schematic of the cavity formation and amnion tissue differentiation on a chip. Abbreviations: iPSC: induced pluripotent stem cell.

Forty-six studies reconstituted the physiological and pathological statuses of organs using the microfluidic chips, including the vagina (n = 2) 113, 114, cervix (n = 6) 115-120, oviduct (n = 5) 121-125, endometrium and decidua (n = 5) 126-130, placenta-decidual interface (n = 15) 131-145, fetal membrane-decidual or choriodecidual interface (n = 8) 146-153, and multi-organs (n = 5; Table 6) 154-158. Two vagina-on-chips delved into the behavior of bacteria 114, and the formation of fungal biofilm 113 through vaginal epithelial cells. One microfluidic chip studied the cervical transitional zone 117. Three cervix chips focused on the reconstitution of the cervical mucus components 115, 118, 122, and one mimicked the geometrical configuration 120. Interactions between embryonic components and decidua were investigated in three chips 127, 128, 130. Among the 15 placenta-on-a-chip studies, five explored placental barrier membrane permeability similar to the *in vivo* condition (Figure 7A) 131, 132, 134-136, and two models explored the placental transferability when exposed to environmental toxins and infection 139, 140. Additionally, two studies focused on placental development 137, 138, and two tested the effects of inflammatory factors 133, 140. Commercial microfluidic chips, such as IFlowPlate (Figure 7B) 141, and three-lane OrganoPlate (Figure 7C) 139, 142 have been adopted. Researchers in the University of Texas Medical Branch at Galveston stand out for their prolific work in constructing fetal membrane-decidual interface microfluidic chips (n = 7), and have extensively studied the effects of oxidative stress (n = 3) 146, 147, 153, and infections (n = 3; Figure 8A) 148, 151, 152. Especially, Yin *et al.* established a human induced pluripotent stem cell-derived three-dimensional (3D) amnion tissue model on a chip to investigate the intra-uterine inflammatory responses 149. Integrating multiple organs on a chip is an ideal approach to investigate the bidirectional crosstalk. Xiao *et al.* fabricated a microfluidic chip featuring the ovary, fallopian tube, endometrium, and ectocervix to stimulate the 28-day menstrual pattern 154. Tantengco *et al.* constructed the vagina-cervix-decidua interface to reveal pathogen infection mechanisms 156. Communications between the ovary and fallopian tube 158, as well as the ovary and uterus have also been studied 155. One study recapitulated the prototype of polycystic ovary syndrome in ovarian and oviductal tissues 157. These innovative approaches have paved the way for a deeper understanding of the organ communications in the physical and pathological conditions.

### Toxicology and drug screening

A total of 14 studies were conducted to assess toxicology or drug efficacy using microfluidic devices (Table 7) 159-172. All papers were reported within recent years, indicating a notable increase in research interest in this field. Only four of these studies employed human samples for experimental purposes 168, 169, 171, 172. The two uterine chips were evaluated with Levonorgestrel 159, and insulin 160. Placental chips were tested using drugs (n = 4) 161, 163, 166, 168, nanoparticles (n = 2) 162, 165, and pollutants (n = 2) 164, 167. A fetal membrane microfluidic chip was utilized to identify the function of a transporter protein in transporting Rosuvastatin 169. Notably, Richardson *et al.* investigated the drug transport and metabolism on both placenta and fetal membrane chips 170. The two ovary-on-a-chip models investigated the effects of Doxorubicin 171, and epidermal growth factor inhibitor 172, respectively.

### Fabrication methods and materials

Polydimethylsiloxane (PDMS; n = 91) was the most often used material, followed by polymethyl methacrylate (PMMA). The fabrication methods corresponded to the materials used. The top methods were soft lithography (n = 67), photolithography (n = 11), and 3D printing (n = 6). Eight studies utilized commercial products.

## Discussion

The use of microfluidic chips has significantly transformed basic and clinical research by replicating multicellular architectures, organ crosstalk, and microenvironmental cues. While several recent reviews have outlined their applications in obstetrics and gynecology field, particularly in the assisted reproductive technology (ART) area 6, 173, 174, there has been no investigation into their use across the entire FRS. Thus, this paper represents the first systematic review to comprehensively delve into the multifaceted functions of microfluidic chips from the female reproduction standpoint. We primarily discuss five aspects of the microfluidics: oocyte handling, embryo manipulations, placenta bioengineering, organ communication, and fabrication methods.

### Using microfluidic chips in ART laboratories

ARTs have revolutionized infertility treatment over the last three decades, enabling millions of individuals to conceive and have children. Annually, more than 2.5 million ART cycles are performed, leading to the birth of over 6.5 million babies worldwide 175. However, the high costs, time-consuming procedures, and labor-intensive efforts limit its utilization 176. Moreover, gametes and embryos are exposed to an external environment during traditional ART procedures, which may increase the risks of fetal malformations, epigenetic disorders, and adverse obstetric or perinatal outcomes 177-179. Therefore, it is essential to provide female reproductive tract cells with a suitable operating environment.

Physical cues in the microenvironment are important yet often overlookded factors. The mechanically heterogeneous extracellular matrix surrounding cumulus cells and oocytes is closely related to follicular growth 180. A stiff ovarian cortex microenvironment is beneficial for maintaining primordial follicles in a quiescent state, while ovarian fragmentation facilitates primordial follicle loss 181, 182. These findings suggest that loading ovary-on-a-chip with controllable degradable biomaterials may restrict follicle growth and achieve sequential activation. In the uterus, mechanical stresses are found to interact with the early embryonic development, thus facilitating pregnancy. Therefore, adjusting shear stress via tilting the embryo culture dish 183, or coating the substrate with collagen has been shown to enhance embryo development and blastocyst formation 184. Despite only a limited number of microfluidic chips have currently improved their performance through modifying mechanical properties 20, 142, utilizing physical cues will greatly enhance the value of the microfluidics. Matrigel, PDMS, collagen, and fibronectin are commonly used extracellular matrix materials 184. It is also recommended to test the stiffness of the extracellular matrix beforehand, and precoat microchambers with materials of similar hardness to mimic *in vivo* conditions when culturing cells derived from these tissues.

In terms of the gamete evaluation, general parameters, including structure, maturity, and oxygen levels, are inefficient at detecting subtle disturbances. With the help of microfluidic chips, highly accurate and reproducible *in situ* assessment can be achieved 21. Other inherent advantages involve the small volumes required, and the integration of various elements in portable devices 75, 79, 88. These chips can quantitatively measure follicular development, assess ovulation disorders, and detect gene and protein expression, as well as metabolic characteristics of endometrial and ovarian cancer cells 20, 185, 186. Indeed, the FRS is understudied when compared with the brain science, bone bioengineering, and the male reproductive system. The microfluidic chips have great potential to revolutionize the diagnosis, and treatment of gynecological and obstetric diseases.

### Cell surgery for oocytes and embryos

The modern medical field has experienced a transition from the utilizing large machines to adopting smaller instruments, which significantly enhances the capabilities of minimally invasive surgeries, drug delivery, and diagnostics. Despite this shift, the manipulation and intervention in single cells remains a notable challenge. On this condition, the microfluidic device has emerged as a powerful tool for conducting cell surgeries, offering unique advantages in accurate manipulation, reduced operation time, a shorter learning period, and the exploration of underlying mechanisms.

Cell surgery involves various intricately complex and highly skilled procedures, including cell position, movement, rotation, and enucleation. Single oocytes are commonly manipulated using microneedles and micro-tweezers, which are housed within microfluidic chips. The microtools can be adjusted via manual, optical, electric, magnetic, or acoustic methods to accurately position the spindle, polar body, and inner cell mass (Figure 5F) 56, 69, 70. The proper positioning of these structures is correlated with higher rates of fertilization and embryonic development 187. Moreover, an alternative method for cell manipulation known as electrowetting-on-dielectric can be utilized to move oocytes (Figure 6C) 64. Cell rotation, on the other hand, can be achieved by activating an electric field, acoustic waves, and magnetic force (Figure 5D) 59, 62, 69. However, the electric field typically results in non-uniform rotation and is challenging to control in and out of the plane. To address this, microstructures are integrated within chips to allow for the rolling of oocytes through oscillating solid microstructures (Figure 5E) 62. Enucleation, a crucial step in cloning efficiency, involves the removal of the nuclear material from mature metaphase II oocytes. Conventional enucleation methods often involve the use of fluorochromes, ultraviolet light, and chemicals, which may cause varying degrees of damage to the cytoplasm 188. The microfluidics may conduct enucleation through magnetic field-controlled microtools. It takes less time, but achieves comparable success rates with manual operation 60, 65, 66. Similarly, cellular cytoplasm can be removed using a micro-knife after an oocyte is grasped with a microgripper 69.

Although automated microfluidic chips for cell surgeries offer many advantages, it is imperative to rigorously examine the potential damage caused by the electric field and optical intensity. Increased laser power from optical devices is shown to disrupt the pH regulation function of cells 189. The electric field has also been reported to damage the cellular membrane and cytoskeleton, ultimately leading to cell death 190, 191. Conversely, a static magnetic field enhances the embryo cleavage and blastocyst formation rates, as well as modulates pluripotency in blastocysts during cumulus-oocyte complexes vitrification 192. The magnetic field induced oocyte damage is associated with the developmental stage 193. Low-intensity electromagnetic fields can disturb oocyte maturation and impact embryo development 194. Prevalent challenges encountered in dielectrophoresis devices encompass Joule heating, bubble generation, insulation breakdown, and undesired reactions occurring on the electrodes, which may adversely affect the microchip function. In light of these findings, additional fundamental evidence is warranted to further investigate the intensity and frequency of confounding factors.

### Bioengineering for placental and fetal membrane research

Medicinal drug usage during pregnancy is imperative in certain scenarios. Nevertheless, due to the fact that pregnant women are often excluded from clinical trials, the risk of these drugs on fetal development remains largely unknown. Moreover, ethical limits and legal concerns also limit placental research, particularly at early stages 195. The advent of microfluidic technology has propelled the creation of the placenta and fetal membrane organ-on-chip models 196. They offer significant potential in investigating embryotoxic effects of drug metabolism and transportation. Among the included studies, glucose is often used as a model substance to test the physiological biomolecular transport 136, 139. These placenta-on-chip models demonstrate superior capability in replicating glucose transport rates, when compared to the Transwell method. Furthermore, studies have introduced various drugs, such as Naltrexone, Glyburide, and statins, into the maternal micro-channels, subsequently measuring the time-dependent changes in the fetal side 132, 135, 166. The effects of nanoparticles (such as titanium dioxide, polystyrene etc.) on trophoblast cell activity or early embryonic development are also topics of interest 162, 165.

Studying pregnancy complications, like preeclampsia and intrauterine growth restriction, poses significant challenges. Animal models often fail to accurately replicate the intricate physiological alterations that occur in human organs during pregnancy. In mouse placenta, there is no separation by the endothelium between the trophoblast and maternal circulation 4. On the contrary to the remodeling of the maternal spiral arteries, the arterial remodeling in rats and guinea pig involves in the endovascular trophoblast cells. The microfluidics technique enables the simulation of *in vivo* placenta and fetal membrane, and has been adopted to study bacterial infection and preeclampsia of placenta. The innate immune alteration, and inflammatory cytokines production caused by *Escherichia coli*. were reported 145. The inflammatory and hypoxic environment acts as an underlying mechanism of preeclampsia. Therefore, tumor necrosis factor alpha and 1%-3% oxygen were employed to mimic the development of preeclampsia 137, 142. Taken together, the utilization of microfluidics will significantly enhance the capacity to mechanistically comprehend the maternal-fetal interface.

### Recapitulating organ communication

In multicellular organisms, organ communications are coordinated through the delivery of bioactive molecules or neurotransmitters via the circulation or neurons, respectively 197. For instance, in response to meal ingestion, the gastrointestinal tract produces gut peptides, which act on the vagal afferent neurons to inhibit food intake and stimulate the liver to metabolize and store glucose 198. Likewise, the FRS involves multiple communications with extra-gonadal organs, with the hypothalamus-pituitary-ovarian axis being a typical core. Additionally, circadian oscillators in the central nervous system also regulate steroidogenesis and oocyte development 199, 200. It was found that gut-derived interleukin-22 improved the disease phenotype in polycystic ovarian syndrome patients 201. Therefore, a comprehensive understanding of the organ crosstalk is crucial for deepening the knowledge of the FRS and developing novel therapeutic approaches.

Conventional tools used to study inter-organ communications include the Transwell system, coculture, and animal models. Despite being an improvement over traditional 2D culture, they face challenges in available tissue types and imaging. In this case, microfluidic approaches serve as a promising alternative to coculture multiple cell-types and tunable perfusion conditions. In the context of the FRS, microfluidic chips allow for the growth of ciliated and secretory cells with correct spatial organizations 125. Researchers have successfully fabricated well-defined placental barriers within microfluidic systems by culturing human umbilical vein endothelial cells (HUVECs) and the b30 clone of the BeWo choriocarcinoma cell line (BeWo b30) on a semipermeable membrane (Figure 7). Most importantly, multi-organ microfluidic chips comprising of the ovary, uterus, cervix, and even liver have been achieved, highlighting the potential of microfluidic platforms in overcoming the limitations of conventional tools for studying inter-organ communications 128, 138, 140, 202.

### Fabrication techniques and materials

The revolution of microfluidic technology and chip fabrication has been advanced by the rapidly developing manufacturing methods, including soft lithography, photolithography, injection molding, laser ablation, and other direct manufacturing technologies 203. Soft lithography emerges as the most commonly used method due to its simple yet robust microchannel manufacturing process, varied pattern options, and high optical transparency (Figure 2). However, the high cost of the equipment renders the technology impractical for large-scale production of microfluidic devices 204. Photolithography offers a high wafer production capacity and is suitable for microscale characteristics. Creating a biomimetic ovarian microstructure through this method facilitates the understanding of ovarian diseases and serves as an *in vitro* culture system for preserving fertility 20. Injection molding offers the advantage of easily manufacturing complex geometric shapes with short production cycles. However, the fabrication of large undercut geometric shapes presents challenges due to potential pattern deformation and the introduction of defects 205. In contrast, laser ablation presents a rapid and scalable method for producing microfluidic devices, but its application is limited by the use of uncommon materials 206. This was exemplified in the fabrication of a microfluidic device with multi-channels using laser ablation of poly (methyl methacrylate) and poly (ethyl phthalate) plastic laminates, which was applied for the detection of specific DNA sequences and a non-specific binding control related to breast and colorectal cancers 207.

When manufacturing microfluidic chips, several characteristics of the raw material must be taken into consideration, like the durability, ease of use, transparency, biocompatibility, and the potential to meet surface functionalization needs. In the early days, microfluidic chips were made from glass, silicon, metals, and ceramics. However, the frequent use of chemical etching to customize the mask undoubtedly increases the time, cost, and effort 208. Currently, the most commonly used polymers are PDMS, PMMA, fluoropolymers, polyurethane, and polycarbonate 209. PDMS, an elastomer with excellent microchip manufacturing performance, is both cheap and easy to mold with good molding outcomes, optically transparent, permeable, biocompatible, low autofluorescence, naturally hydrophobic, and highly elastic 210. However, when employed in cell culture substrates, PDMS tends to adsorb hydrophobic molecules, resulting in reduced drug concentration and activity 89. An appealing alternative is polyurethane elastomer. It possesses similar optical transparency, flexibility, and castability as PDMS, but notably, it is resistant to the absorption of small hydrophobic molecules. PMMA exhibits superior solvent compatibility compared to PDMS, and its lack of small-molecule absorption also renders it a favorable material for microchip fabrication 211. These characteristics are particularly useful for on-chip organ devices and micro-physiological systems. There is ongoing exploration of many other suitable polymers for microfluidic chip fabrication.

The hydrogel is one of the important compartments in constructing microfluidic chips, and has been widely used in the FRS. The cervical mucus serves as a liquid barrier to prevent infection, and screen sperm. To mimic the cervical microenvironment, hyaluronic acid hydrogels have been employed as a surrogate, and share similarity in viscosity and charge with cervical mucus 118. However, hyaluronic acid may compromise the cellular viability, and methylcellulose hydrogels are preferred by some groups 122, 212. Extracellular matrix hydrogels containing collagen type I and IV are usually used to create* in vitro* placenta, and fetal maternal interface models 131, 139. In these cases, the fetal and maternal sides locate on either side of the hydrogel microchannels. Less frequently used hydrogels, including methacrylamide 134, 137, laminin 135, and Matrigel 135 have also been adopted in some studies.

### Features of 3D-printed microfluidic chips

Three-dimensional printing has revolutionized the fabrication of high-precision microfluidic chips, for its characteristics in flexibility, directness, and rapid prototyping 213. It overcomes the disadvantages of soft lithography and other traditional miniaturization methods, such as the inability to create a true 3D structure, the costly and time-consuming design process, and the challenges in batch manufacturing 214. Moreover, the use of high printer resolution in 3D printing has enabled the manufacturing of miniaturized and microfluidic systems with significantly reduced equipment and sample requirements 215. The incorporation of translucent, heat-resistant, and biocompatible materials in 3D printing allows for its future applications in biotechnology.

Among the various 3D printing technologies, fused deposition modeling, inkjet 3D printing, and photopolymerization obtain much popularity due to their well-established procedures and cost-effectiveness with respect to equipment and materials 214. Fused deposition modeling is characterized by its cost-saving and widespread availability for processing thermoplastics. However, its low accuracy level and nozzle clogging result in mechanically weak devices 216. On the other hand, inkjet 3D printing offers improved accuracy, and produces multi-material/colored objects with high surface precision. Nonetheless, issues such as poor material durability and low mechanical strength limit its application 217. Photopolymerization, which encompasses technologies such as stereolithography and digital light processing, stands out for its exceptional accuracy and resolution, making it ideal for constructing complex structural devices with smooth surfaces and versatile printed parts 218. Nevertheless, concerns related to the compatibility, biocompatibility, and toxicity of the printing resins need to be addressed, particularly in the context of bioprinting. As a whole, the search for a wide range of materials suitable for bioprinting continues to present a challenge in this field.

### Future perspectives

#### Use of commercial products

The commercial development of microfluidic chips is a crucial element in advancing organ chip technology. Unlike those used in laboratories, commercial organ chips require careful consideration of cost, yield, structural dimension tolerance, and manufacturability. To address cost concerns, for instance, injection-molding materials may replace PDMS, and the soft lithography manufacturing method may be substituted with injection molding. Altering material surface characteristics can significantly impact product performance and quality. Therefore, it is essential to take mass production factors into account from the early stages of experimental research. This proactive approach can lead to cost and time reduction in transitioning microfluidic chips into commercial products.

Advancements from the laboratory to commercialization are primarily carried out through start-ups that attract investments to transform the chip-organ industry. At present, several companies produce microfluidic chips and related commercial products. Hesperos has combined lab expertise to develop organ chips for clinical applications. Multi-organ analysis systems with built-in mechanical and chemical biosensors have been produced to tackle rare diseases by a human-on-a-chip approach. Hesperos' key technology is a pumpless four-organ (heart, liver, neurons, and skeletal muscle) system, where toxicology and functional responses of five drugs are clinically diagnosed and analyzed 218. The start-up company TissUse, established in Germany, is developing two-organ and four-organ microfluidic chips by integrating biological vasculature into a multi-organ chip microsystem. These chips have an open structure, allowing tissues to be externally assembled and prepared before being placed into the device cavity after assembly. Such an approach is compatible with clinically relevant tissue biopsies 219. ChipSensors focuses on developing a technology to support microfluidic chip-based diagnostics and life science research. Microfluidic-Tech provides various microfluidic chips, ranging from polymerase chain reaction to reagent detection chips. Microfluidic Chip Systems has patents related to microfluidic technology, and their products include microfluidic chips and solutions for biological analysis. Microfluidics UK provides high-performance microfluidic chips and equipment for microfluidic analysis applications such as molecular biology, cell biology, and materials science. Finally, Nordic Microfluidics produces easy-to-use, time-saving, and cost-effective microfluidic chips, helping customers solve microfluidic analysis challenges. The miniaturization, integration, functionalization, and intelligence of the whole organ chips should be considered in clinical translation to expand to individualized medical treatments.

#### Toward an all-in-one microfluidic chip

**Figure 9:**
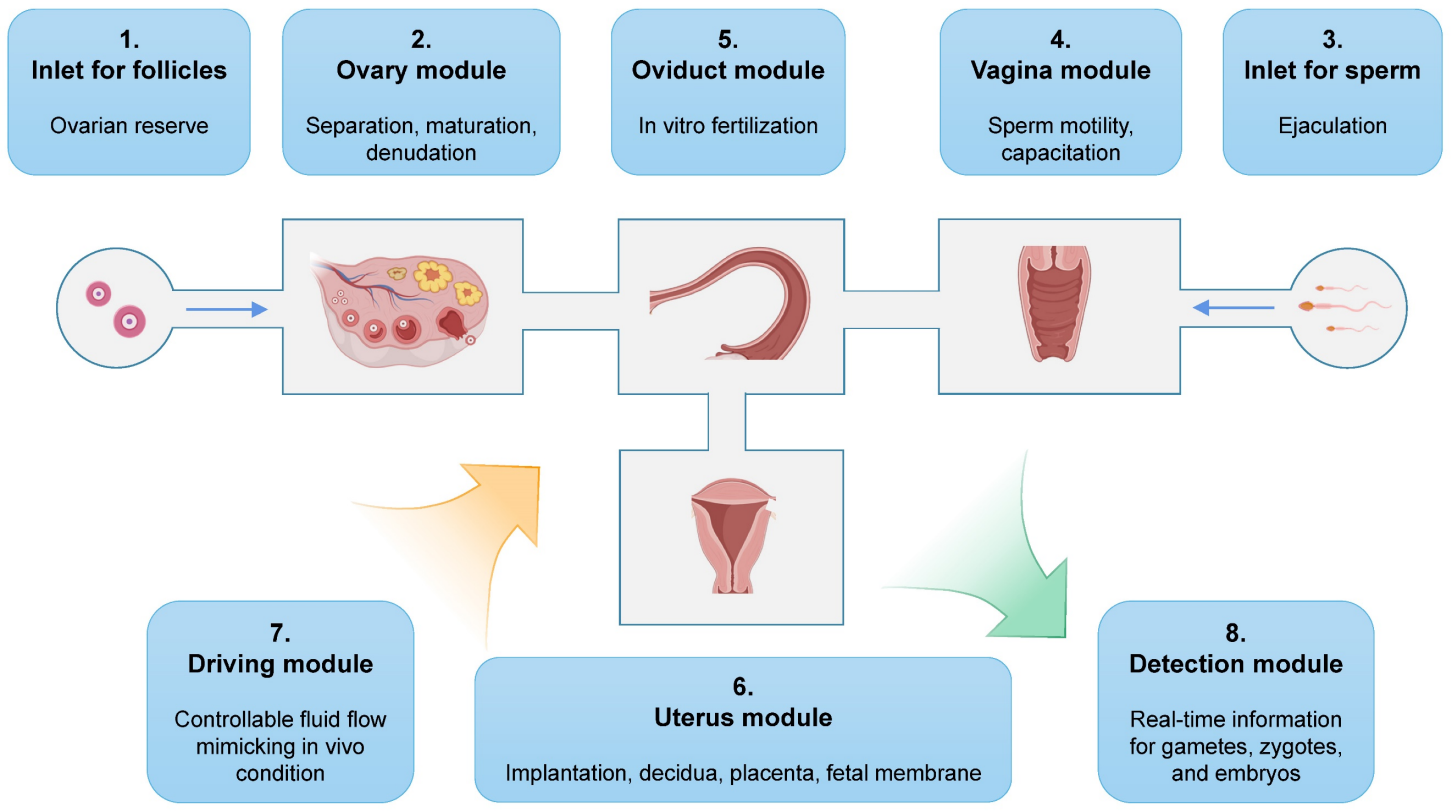
An example of all-in-one microfluidic chips that mimic the entire female reproduction tract.

The development of a fully functional microfluidic chip that contains a complete FRS presents an intriguing prospect. Operators would simply need to load eligible cumulus-oocyte complexes into the device, and the all-in-one microfluidic chip is capable of automating oocyte manipulation, fertilization, and early embryo culture (Figure 9). After a few days, well-developed embryos would be obtained. The potential of this technology is highlighted by Xiao *et al.*, who developed the EVATAR microfluidic chip containing female reproductive tract explants and peripheral tissues 154. Their findings demonstrated the reproducibility of a coordinated response to estrogen and progesterone using the microfluidic chip technology. Similarly, Park *et al.* fabricated a microfluidic device incorporating human endometrial and primary ovarian somatic cells, revealing the facilitation resulting from bidirectional endocrine crosstalk, as well as identifying a predictive marker of reproductive toxicity 155. It is important to note that each chamber should be designed to support specific functions for each organ, rather than serving as simple supporting platforms. For instance, the spatial separation of theca and granulosa cells could contribute to optimized hormone secretion, while the microchannels could be filled with hyaluronic acid to simulate cervical viscosity 220. Though the goal of achieving an integrated system is challenging, it represents a substantial opportunity for further advancements in reproductive technology.

Integration of laboratory testing into all-in-one microfluidic systems represents another significant advancement. Conventional hormonal tests typically involve antigen-antibody binding and signaling magnification, which are usually conducted in centralized laboratories with specialized instrumentation and personnel, resulting in low throughput and imposing a heavy burden on medical facilities. However, recent advancements in microfluidic chip technology exhibit promising performance characteristics, such as simultaneous detection, rapid treatment, low consumption, high throughput, and miniaturization. For instance, Lee *et al.* integrated droplet and bead manipulation together, and detected two growth factors from a single embryo with only 520 nL in less than 40 min 108. More specifically, the range of testing has been extended to mechanics, cell permeability, viability, and oxygen consumption. To optimize the biomedical and clinical diagnosis, it is essential to integrate the aforementioned components into a fully functional microfluidic chip. By harnessing the power of multiplex detection for reproductive disorders, it is possible to reduce the likelihood of misdiagnosis and incomplete diagnosis of diseases with overlapping symptoms, thereby enhancing the overall efficiency of medical testing procedures.

#### Selection of human cells

Since animal research findings are typically extrapolated to humans, suitable human cell components should be employed to bring the *in vitro* systems to the closest possible similarity to the physiological environment. Although the overall utilization rate of human samples reached 58.3%, most studies were limited to pregnancy-related research and only sparsely investigated other organs or tissues. In fact, the human and mouse female reproductive tracts differ significantly 221. Macroscopically, the gross uterine anatomy of reproductive-age humans is pear-shaped, while the one in mice is Y-shaped. Microscopically, the human cortical region occupies a thin layer of <1 mm in the outmost part of the ovaries 222, while the mouse cortex takes up most of the ovarian volume. The mouse estrous cycle is around 4-5 days with no vaginal bleeding, while the menstrual cycle in humans is around 28 days. Researchers must be aware of the similarities and divergences across species to facilitate clinical translation 223.

Human placental cells are always preferred because they are easily accessible and devoid of ethical issues. A wide range of placental cells, including HUVECs, human placental villous endothelial cells, and several cell lines (such as BeWo b30, HTR8/SVneo, CS2, JEG-3 etc.), have been utilized. In the included studies, HUVECs are the most extensively used endothelial cells. They are well characterized, convenient to identify, as well as relatively easy to obtain and culture 132. BeWo b30 cells are widely utilized in research on placental permeability, as they possess the unique characteristic of not undergoing contact inhibition 224. This feature allows them to form multilayers following a one-week culture period, which is important for ensuring the integrity of the biomimetic membrane 225. Despite the above advantages, these cells may not be the best source due to their choriocarcinoma origin. For example, the primary syncytiotrophoblasts are insensitive to glucose transport inhibition, while BeWo b30 cells show a proportional alteration in transepithelial transport after the treatment 226.

Human trophoblast stem cells (TSCs) have introduced a novel method for replicating placental development in the first-trimester. They can be isolated from human blastocyst outgrowths or primary placentas 227. The TSCs are capable of long-term proliferation *in vitro*, and are functionally competent to differentiate into syncytiotrophoblasts and extravillous cytotrophoblasts 227. The human TSCs can also be derived from human pluripotent stem cells 228, or somatic cells via direct reprogramming 228, 229. Furthermore, generation of induced trophoblast stem cells is also feasible 229. Taken together, stem cell technology provides an accessible and patient-specific model system of human placental research. It will greatly facilitate the study of placental biology and diseases, and their treatment.

Human oocytes and embryos are needed to address important scientific questions. Researchers should redesign the culture chambers' size and choose an appropriate medium when shifting from experimental animals to humans. However, human samples are difficult to obtain, resulting in the small number of studies that used human oocytes and embryos. Most importantly, full informed consent should be recorded 230. Fortunately, one advantage of microfluidic chips is the small number of cells needed for culture. It is essential to consider human cell selection when designing experiments to construct models of the female reproductive system.

## Limitations

This systematic review had some limitations. First, the eligible studies lacked rigorous interpretation of the risk of bias because most studies focused on the engineering process. Information on animal randomization, blinding, and biological replicates were not provided. Therefore, we provided detailed information about microfluidic chip designs and biological assessments to fill this void. Then, due to the high technical threshold, the included data relied heavily on small-sample studies, particularly those on vaginal, uterine and oviductal chips, which could potentially decrease the applicability of the findings to other research groups and laboratories. Possibly for the same reason, analysis of drugs, toxins, and chemicals in the female reproduction field was also scarce.

## Conclusion

Recreating the organ function of the female reproductive tract and promoting clinical applications are complex and challenging. The microfluidic chip emerges as an enhanced novel technical support to help achieve this goal. This review highlights the feasibility of microfluidic chips in oocyte operation, embryo manipulation, organ stimulation, and drug screening. As microfluidics is developing rapidly, future research should focus on the development of novel materials and the creation of multifunctional devices.

## Supplementary Material

Supplementary table.

## Figures and Tables

**Table 1:** *In vitro* culture and maturation of oocytes using microfluidic chips.

Reference	Key group	Main finding
Oocyte		
Choi *et al.* (2014)	① Dish ② Chip + hydrogel encapsulation	Using alginate (harder) and collagen (softer) to investigate the mechanical heterogeneity during follicle development and ovulation.
Zargari *et al.* (2016)	Descriptive study without grouping.	Trapping oocytes and tracking the maturation process.
Aziz *et al.* (2017)	① Dish ② Chip	Similar hormonal trends and similar diameters in ①②.
Berenguel-Alonso *et al.* (2017)	① Dish ② Chip	No significant differences in nuclear and cytoplasmic maturation in ①②.
Huang *et al.* (2018)	Electric field activation with various duration and intensity.	Higher alternating current electric field improves the parthenogenesis of oocytes without sperm insemination.
Nagashima *et al.* (2018)	① Dish ② Chip + static flow ③ Chip + various dynamic flow	No significant differences in follicle development markers among ①②③.
Healy *et al.* (2021)	① 2D culture of ovarian somatic cells ② Chip + encapsulation	Higher androstenedione and lower progesterone in ② than ①.
Sadeghzadeh Oskouei *et al.* (2021)	① Chip + static flow ② Chip + passive flow ③ Chip + active flow	Lower malondialdehyde concentration and fewer apoptotic cells in ②③ than ①.
Del Valle *et al.* (2022)	① No treatment ② Chip + static flow ③ Chip + slow flow ④ Chip + dynamic flow	More apoptotic stromal cells in ④ than ①②③. Increased collagen deposition in ②③ than ①.

**Table 2:** Applications of the microfluidics to observe and evaluate oocytes/embryos.

	Reference	Main finding
**Oocyte**		
**Mechanics**	Liu *et al.* (2010)	Distinguishing young and old oocytes real time through injection force measurement.
	Arai and Sakuma (2015)	High-throughput oocyte mechanical characterization using a magnetically driven on-chip probe.
	Nakahara *et al.* (2015)	Simultaneous transportation and mechanical measurement of oocytes through a micropillar array in an open environment.
	Nakahara *et al.* (2018)	The Young's modulus of the zona pellucida of cryopreserved oocytes increases with cultivation time.
	Andolfi* et al.* (2019)	Quantitative estimate of the relaxation times of oocytes using planar atomic force microscopy macro-probes.
	Pokrzywnicka *et al.* (2019)	Oocyte quality classification according to the deformation change.
	Saffari *et al.* (2023)	Abnormal cortical tensions in oocytes lead to unsuccessful growth, and 79% of oocytes with tensions between 1.5 and 3 nN/μm reach the metaphase II stage.
	Azarkh *et al.* (2023)	Utilizing transient electrical impedance spectroscopy to detect the viscoelastic properties of zona pellucida.
**Permeability**	Zhao *et al.* (2017)	Characteristics of oocyte permeability under different cryoprotectant and temperature conditions.
	Chen *et al.* (2019)	High- and poor-quality oocytes are classified based on the membrane permeability.
	Lei *et al.* (2019)	Testing the membrane permeability of oocytes exposed to various cryoprotectants at different temperatures.
	Chen *et al.* (2020a)	Analysis of multiple oocytes volume under different types cryoprotectants and continuous concentration change.
	Guo *et al.* (2020)	Testing oocyte volume under different types and concentrations of cryoprotectants.
	Tu *et al.* (2022)	Monitoring the osmotic response of oocytes using the experimental and computational techniques.
**Structure**	Angione *et al.* (2015)	Real-time, longitudinal imaging of oocytes following fluorescent labeling.
	Luo *et al.* (2015)	Evaluating the spindle of oocytes via a constricted microfluidic channel.
	Podwin *et al.* (2020)	Evaluating the expansion ability of oocytes.
**Spectrum**	Sniadek *et al.* (2011)	Dividing oocytes into two classes under microspectrometry.
	Walczak *et al.* (2012)	Dividing oocytes into large, middle, and small groups using microspectrometry.
	Gorecka-Drzazga (2012)	Testing the optical signals of fluorescent-marked oocytes.
**Electricity**	El Hasni *et al.* (2017)	Higher impedance for zona pellucida-free oocytes than for those with intact zona pellucida.
	Cao *et al.* (2023a)	Calculating the Young's modulus of zona pellucida based on the micropipette aspiration technique.
**Oxygen**	Tedjo *et al.* (2021)	Analysis of oxygen consumption rate and oxygen flux density of cumulus-oocyte complexes via an electrochemical sensor.
**Embryo**		
**Structure**	Jang *et al.* (2010)	Observations via phase contrast imaging, double interferogram, and optical path length.
	Bae *et al.* (2011)	The pressure and duration of applied mechanical stimulus compromise embryonic growth.
	Vandormael-Pournin *et al.* (2021)	An eggbox imaging device designed to observe preimplantation embryos.
	Mohagheghian *et al.* (2023)	Greater tensile and compressive traction force oscillations than shear traction force oscillations in mouse blastocyst.
**Viability**	Śniadek *et al.* (2012)	Utilizing miniaturized fluorescence to discriminate between non-apoptotic and apoptotic embryos.
	Sivelli *et al.* (2022)	Using nuclear magnetic resonance technology to observe single mammalian embryo for embryo transfer.
**Oxygen**	Date *et al.* (2011)	The oxygen consumption increases from morula to blastocyst.
	Kurosawa *et al.* (2016)	Estimating the oxygen consumption rate of spheroids, bovine embryos and frozen-thawed human embryos, and it corresponds to the developmental potential of embryos.
**Hormone**	Heo *et al.* (2012)	Analysis of embryo glucose production using a mechanical deformation-based actuation.
	Chen *et al.* (2020b)	Combination of microfluidic droplets and multicolor fluorescence to accurately detect hCG-β at single embryo level.
	Lee *et al.* (2020)	On-chip immunoassay for human interleukin-1β and TNF-α to distinguish the developmental embryos.

Abbreviations: hCG: human chorionic gonadotropin; TNF: tumor necrosis factor.

**Table 3:** Oocytes and embryos cryopreservation using microfluidic chips.

Reference	Group	Main finding
**Oocyte**		
Heo *et al.* (2011)	① Chip + step-wise ② Chip + linear ③ Chip + complex	Complete equilibration after cryoprotectants loading in <15 min, and with <10% oocyte volume changes.
Yang *et al.* (2012)	Different constant cooling rates	Slow freezing is feasible for cryopreservation of germinal vesicle porcine oocytes.
Lai *et al.* (2015)	① No treatment ② Manual ③ Chip + gradual addition ④ Chip + abrupt sucrose ⑤ Direct addition	Higher cytoplasmic lipid retention, less cytoplasmic leakage and higher developmental competence in ③ than ②.
Zhou *et al.* (2018)	① Manual ② Chip	Higher survival rate and cleavage rate of frozen oocytes in ② than ①.
Guo *et al.* (2019)	① No treatment ② Manual + step-wise ③ Chip + linear	Less osmotic damage of oocytes in ③than ②.
Shao *et al.* (2019)	Different loading times, line types, and concave loading types	Novel parameters and entropy methods are proposed and are highly negative correlated with blastocysts rate.
Zhou *et al.* (2019)	Linear/convex/concave loading/unloading of CPA	The concave loading-convex unloading protocol shows the highest survival rate and morula rate.
Miao *et al.* (2022a)	① Manual ② Chip	Comparable survival rate, mitochondrial membrane potential and reactive oxygen species levels in ①②.
**Embryo**		
Pyne *et al.* (2014)	① No treatment ② Manual ③ Chip	Embryo survival and development rates are comparable in ②③.
Tirgar *et al.* (2021)	① Manual ② Chip	Comparable re-expansion, hatching rates, and reduced detrimental gene expression levels in ①②.
Miao *et al.* (2022b)	① No treatment ② Manual ③ Chip	Comparable survival rates and development rates in ①②.
Miao *et al.* (2023)	① Manual ② Chip	A satisfactory success rate, and high quality of vitrified embryos after thawing in ②.

**Table 4:** Applications of microfluidic chips to observe or manipulate oocytes.

	Reference	Main finding
**Separation**	Kawahara *et al.* (2012)	Dispensing single oocytes through air-flow based inkjet mechanism.
	Feng *et al.* (2013a)	A single enucleated oocyte dispensing system with a 100% success rate, and a 70% survival rate.
	Iwasaki *et al.* (2018)	Separating high-quality oocytes based on sedimentation rate differences for further *in vitro* fertilization.
	Uning *et al.* (2020)	Combination of a rack-pinion-based loader and a bubble injector to separate single oocyte with a >80% success rate.
**Rotation**	Hagiwara *et al.* (2010)	Precise positioning of oocytes by horizontally arranged permanent magnets with two degrees of freedoms.
	Hagiwara *et al.* (2012)	Attraction, repulsion, and rotation of oocytes are conducted by adjusting the oscillation parameters of micro-tools.
	Benhal *et al.* (2014)	Three-dimensional cell rotation on an alternating current induced electric field-based platform.
	Hayakawa *et al.* (2015)	Circular vibration induced by a micropillar via a piezoelectric actuator to rotate oocytes.
	Feng *et al.* (2016)	Three-dimensional rotation of a single oocyte with an accuracy of one degree and an average rotation velocity of three rad/s.
	Feng *et al.* (2018)	Trapping and rotation of oocytes using oscillating asymmetrical microstructures with different vibration modes.
	Feng *et al.* (2019)	Precise rotation from in-plane and out-of-plane using acoustic microstreaming generated by oscillating asymmetrical Microstructures.
	Bai *et al*. (2020)	Tunable trapping and rotation of oocytes in an acoustofluidic device.
**Enucleation**	Clow *et al.* (2010)	Automated cell positioning by dielectrophoresis and nuclear transfer by electrofusion.
	Hagiwara *et al.* (2011)	High-throughput enucleation with two magnetically driven microtool blades.
	Inomata *et al.* (2011)	Cutting an oocyte using the magnetically driven four-leg-type configuration microtool.
	Ichikawa *et al.* (2011)	An automated enucleation by high-precision control using a high-response and high-precision syringe pump.
	Feng *et al.* (2013b)	A microfluidic chip with a magnetically driven microrobot and controlled flow speed for oocyte enucleation
	Ichikawa *et al.* (2014)	A microknife and a microgripper were set to produce enucleated oocytes with a success rate of 100%.
	Liu *et al.* (2020)	Reduced batch process duration, comparable success rate and survival rate when compared to manual process.
**Denudation**	Weng *et al.* (2018)	Denudation through jagged-surface constriction microchannels.
	Mokhtare *et al.* (2022)	Using acoustic streaming and acoustic radiation force to agitate cumulus-oocyte complexes.

**Table 5:** *In vitro* fertilization and culture of primary embryos using microfluidic chips.

Reference	Key group	Main finding
**Embryo (mono-culture)**		
Akagi *et al.* (2010)	① Dish ② Chip	Similar developmental outcomes in ①②.
Sugimura* et al.* (2010)	① Droplet ② Chip + dynamic flow ③ *In vivo*	Reduced apoptosis and increased pregnancy rate in ② than ①.
Han *et al.* (2010)	① Dish ② Chip	Similar fertilization rates in ①②.
Heo *et al.* (2010)	① Dish ② Chip + static flow ③ Chip + dynamic flow ④ *In vivo*	Enhanced blastocyst development and cell numbers in ③ than ①②. Improved embryo implantation and ongoing pregnancy rates in ③ than ②.
Villa *et al.* (2010)	Descriptive study without grouping.	Supporting the highly proliferative status of pluripotent embryonic stem cells.
Ma *et al.* (2011)	① Dish ② Chip	Similar embryo growth rate and blastocyst formation between ① and ②.
Esteves *et al.* (2013)	① Dish ② Chip + static flow ③ Chip + dynamic flow	Improved embryonic development, blastocysts rate, and birth rate in ②③ than ①.
Wang *et al.* (2014)	① Droplet ② Chip + dynamic flow	Higher rates of 5-8 cell embryo rate, morula rate and blastocyst rate in ② than ①.
Huang *et al.* (2015a)	① Chip + static ② Chip + dynamic	Higher rate of embryo cleavage to a hatching blastocyst in ② than ①.
Huang *et al.* (2015b)	① Dish ② Chip	The average rate of fertility is about 52% in ②.
Kieslinger *et al.* (2015)	① Dish ② Chip	Comparable blastocyst development in ① and ②.
Huang *et al.* (2018)	① Dish ② Chip	Higher fertilization rate, and more blastocyst stage in ② than ①.
Li *et al.* (2018)	① Dish ② Chip	Higher rates of development to morulae and blastocysts, and more inner cell mass cells in ② than ①.
Yekani *et al*. (2018)	① Dish + embryo ② Dish + SB ③ Chip + SB ④ Chip + grouped SB ⑤ Chip + grouped SB + embryo	Improved development of mouse SBs in ③④⑤ than ②.
Chiu *et al.* (2019)	Descriptive study without grouping.	A five-day culture includes emulsion, sorting, expansion and restoration in a droplet.
Huang *et al.* (2020)	① Dish ② Chip	Comparable blastocyst rate in ①②.
Hawkins *et al.* (2022)	① Dish ② Chip ③ Chip + pluronic F127 ④ Chip + deionized water	Improved blastocyst rate in ③ than ②④, and comparable apoptosis, DNA breakage and replication in ①②.
Karcz *et al.* (2023)	Descriptive study without grouping.	Utilizing electrowetting on dielectric technology to manipulate bovine embryos *in vitro*
**Embryo (co-culture)**		
Li *et al.* (2013)	① Dish + ESC ② Chip + ESC	Higher four-cell rate, morula rate and blastocyte rate are achieved in ② than ①.
Li *et al.* (2014)	① Dish + OEC ② Chip + OEC	Higher blastocysts formation rate, and more cell numbers in ② than ①.
Chang *et al.* (2016)	① Dish + ESC ② Chip + ESC	The blastocyst rate increases along with the increasing progesterone concentration.
Ferraz *et al.* (2017)	① Dish + OEC ② Chip + OEC	More matured oocytes in ② than ①, and no polyspermy and parthenogenic activation in ②.
Ferraz *et al.* (2018)	① *In vivo* ② Dish + OEC ③ Chip + OEC	More physiological zygote genetic reprogramming in ③ than ②.
Chen *et al.* (2021)	① Dish ② Chip ③ Dish + MSC ④ Chip + MSC	Embryos grow faster, and the blastocyst development rate increased in ④ than ③.
Wang *et al.* (2022)	① Dish ② Chip without cells ③ Chip + OEC	Lower intracellular reactive oxygen species in four-cell stage embryos in ③ than ①.

Abbreviations: ESC: endometrial stromal cell; MSC: mesenchymal stem cell; OEC: oviduct epithelial cell; SB: single blastomere.

**Table 6:** *In vitro* organ reconstitution under physio- and patho-states.

	Reference	Main finding
**Vagina**	Czechowicz *et al.* (2022)	Observation of the biofilm formation of *Candida* on the surface of the vaginal epithelium.
	Mahajan *et al.* (2022)	Effects of co-culturing vaginal epithelium with *Lactobacillus crispatus* and *Gardnerella vaginalis*.
**Cervix**	Zhang *et al.* (2012)	Imitation of the physiological interactions between cervical mucus and sperm.
	Tung *et al.* (2014)	Studying the effects of surface topography and fluid flow in bovine cervix.
	Tantengco *et al.* (2021)	Simulating the interactions between the ectocervical and endocervical epithelial layers.
	Yu *et al.* (2022)	Filling cervix channels with hyaluronic acid to study the viscosity and charge of cervical mucus.
	Leemans *et al.* (2022)	Establishing a functional model that resembles the *in vivo* oviduct epithelium.
	Dadkhah *et al.* (2023)	Serpentine microchannels with different radii of curvature to mimic the tortuous cervical structure.
**Oviduct**	Xie *et al.* (2010)	Chemo-attractive function of cumulus-oocyte complexes in the oviduct.
	Yan *et al.* (2020)	Mixture of human tubal fluid and methylcellulose to mimic the female viscous environment.
	Raveshi *et al.* (2021)	Droplet microfluidics to create soft curved interfaces with different radii of curvature corresponding to the labyrinthine complexity of human fallopian tube.
	Yaghoobi *et al.* (2022)	A microfluidic platform mimicking the structure of lumen in the uterotubal junction.
	Yu *et al.* (2023)	Forming a progesterone gradient in fibronectin-filled microchannels to mimic the oviductal microenvironment.
**Endometrium and decidua**	Gnecco *et al.* (2019)	Stromal decidualization under hemodynamic forces is mediated via cyclooxygenase-2, as well as the paracrine actions of prostaglandin E2 and prostacyclin.
	Govindasamy *et al.* (2021)	Trophoblast giant cells migrate collectively and communicate with endothelial cells during implantation.
	Park *et al.* (2022)	Extravillous trophoblasts directly migrate towards the maternal vessel and interact with decidualized stromal cells for vascular remodeling.
	Liu *et al.* (2023)	Follistatin exerts more significant effects on cellular adhesion, wound healing and migration of decidualized endometrial stromal cells than Activin A.
	Govindasamy *et al.* (2023)	Modeling the endothelial cell-embryo interaction in a methacrylated dextran and polyethylene glycol hydrogel environment.
**Placenta-decidual interface**	Lee *et al.* (2016)	Confirming the roles of trophoblast and endothelial cells in placental barrier function.
	Blundell *et al.* (2016)	Inducing trophoblast cells to form a syncytialized epithelium progressively, and reconstituting the expression and physiological localization of membrane transport proteins.
	Abbas *et al.* (2017)	The granulocyte-macrophage colony-stimulating factor induces the migration of human trophoblast cells.
	Mandt *et al.* (2018)	Fabricating biomimetic placental barriers with optimal structuring parameters, material composition and cultivation conditions.
	Zhu *et al.* (2018)	Employing *Escherichia coli*. to mimic the bacterial infection and observe transplacental communication.
	Pemathilaka *et al.* (2019)	A steady caffeine state is reached in maternal (0.1513 mg/ mL, 6.5 h) and fetal (0.0033 mg/mL, 5 h) sides.
	Mosavati *et al.* (2020)	Glucose diffusion across the placental barrier in different models and flow rates.
	Ko *et al.* (2022)	Enhanced cell invasion of trophoblast cells in a hypoxic environment to study placental development.
	Li *et al.* (2022)	Follistatin induces migration and invasion of trophoblasts through JNK signaling in placental development.
	Mosavati *et al.* (2022)	Infections resist glucose perfusion and decrease the glucose transfer.
	Ghorbanpour *et al.* (2023)	Investigating the role of inflammatory factors in preeclamptic placenta.
	Kouthouridis *et al.* (2023)	Differentiated placental stem cells produce a more highly fused syncytium that is consistent with *in vivo* findings.
	Rabussier *et al.* (2023)	Construction of physical and pathological placental barrier on-a-chip models.
	Cherubini *et al.* (2023)	Rising flow in perfusable fetal microvessels shows improved function and enhanced protein expression in placental vascularization model.
	Cao *et al.* (2023b)	Self-assembled human placental model using trophoblast stem cells in a dynamic system.
**Fetal membrane-decidual or choriodecidual interface**	Richardson *et al.* (2019)	Testing the effects of oxidative stress on amnion membrane organ-on-chip.
	Richardson *et al.* (2020a)	Better membrane permeability regardless of the side of treatment or time point in chips than Transwells when exposed to cigarette smoke extract or dioxin.
	Richardson *et al.* (2020b)	Ascending infection propagates through the chorion, amnion mesenchyme, and reaches the fetal amnion within 72 hours and induces time-dependent and cell-type specific pro-inflammatory cytokine production.
	Yin et al. (2020)	Investigating intrauterine inflammation with human induced pluripotent stem cell-derived 3D amnion tissues.
	Radnaa et al. (2021)	Fetal membrane-derived exosome mediated paracrine signaling generates inflammation and induces parturition.
	Bento *et al.* (2023)	*Ureaplasma parvum* causes limited inflammatory response in the choriodecidual interface, but not in the amnion layer.
	Richardson* et al.* (2023a)	Fetal oxidative stress causes more amnion-based cellular pathologies and inflammation, whereas maternal oxidative stress induces localized immune regulatory changes.
	Richardson* et al.* (2023b)	Lipopolysaccharides induce decidual inflammation, infiltration of NK cells and neutrophils, as well as progesterone production in chorion cells.
**Multi-organs**	Xiao *et al.* (2017)	Simulating the *in vivo* female reproductive tract and the endocrine loops between organ modules for the ovary, fallopian tube, uterus, cervix and liver.
	Park *et al.* (2020)	Introducing the SERPINB2 luciferase reporter system to test the toxicity in uterine endometrium and ovaries.
	Tantengco *et al.* (2022)	*Ureaplasma parvum* infection neither promotes cell death nor causes massive inflammation in the vagina-cervix-decidua interface cells, but the damage increases when combined with lipopolysaccharide or directly inoculates the amniotic cavity.
	Russo et al. (2022)	Ovary-derived versican is released during ovulation to increase activity of fallopian tube epithelium.
	Campo *et al.* (2023)	Recreating the polycystic ovarian syndrome phenotypes using ovarian and oviductal tissues.

**Table 7:** Drug screening and toxicity test conducted on microfluidic chip platforms.

	Reference	Key group	Main finding
**Uterus**	Ahn *et al.* (2021)	① Chip + control ② Chip + Levonorgestrel (10, 100, 1000, and 10000 ng/mL)	More dead cells in ② than ①.
	Baik *et al.* (2023)	① Chip + control ② Chip + vehicle ③ Chip + insulin (10 ng/mL)	Transcriptional comparisons of the endometrial epithelium among ①②③.
**Placenta-decidual interface**	Blundell *et al.* (2018)	① Chip + Glyburide ② Chip without cells + Glyburide	Efflux transporters mediate the active transport function of the human placental barrier.
	Yin *et al.* (2019)	① Chip + vehicle ② Chip + TiO2 (50, 200 μg/mL)	More damage to barrier integrity and impaired immune cells in ② than ①.
	Pu *et al.* (2021)	① Chip + vehicle ② Chip + folic acid (100 ng/mL)	More cell invasiveness in ② than ①.
	Boos *et al.* (2021)	① Chip + embryoid body + polystyrene ② Chip without placenta + embryoid body + polystyrene	Reduced polystyrene concentration in fetal side in ① than ②.
	Abostait *et al.* (2022)	① Static ② Static + forskolin ③ Dynamic ④ Dynamic + forskolin Toxicity: liposome	Increased cell uptake of liposome in ②③④ than ①.
	Pemathilaka *et al.* (2022)	① Chip without cells + Naltrexone (100 ng/mL) ② Chip without cells + 6β-Naltrexol (100 ng/mL) ③ Chip + Naltrexone ④ Chip + 6β-Naltrexol	Less drug enrichment in ③④ than ①②.
	Ticiani *et al.* (2022)	① Chip + control ② Chip + Bisphenol S	Bisphenol S prevents EGF mediated extravillous trophoblasts functions.
	Kammala *et al.* (2023)	① *Ex vivo* placenta perfusion ② *In vivo* murine model ③ *In silico* ④ Chip	Pravastatin transfer in ③④ are similar.
**Fetal membrane**	Ganguly* et al.* (2021)	① Chip + Rosuvastatin + control siRNA ② Chip + Rosuvastatin + OATP2B1 siRNA	Reduced Rosuvastatin propagation from the decidua to the fetal amnion epithelial cell layer in ② than ①.
**Placenta and fetal membrane**	Richardson *et al.* (2022)	① Fetal membrane/placenta Chip ② Fetal membrane/placenta Chip + oxidative stress Drug: Pravastatin and Rosuvastatin (200 ng/mL)	The drugs permeate the maternal-fetal interface chips and generate cell- and time-specific statin metabolites.
**Ovary**	Aziz *et al.* (2020)	① Chip ② Chip + Doxorubicin	Reduced follicular growth and hormone secretion, more apoptosis in ② than ①.
	Lee *et al.* (2022)	① Chip ② Chip + EGF inhibitor	Higher expansion area in ① than ②.

Abbreviations: EGF: epidermal growth factor; OATP2B1: organic anion transporting polypeptide 2B1.

## Abbreviations

2D: two-dimensional
3D: three-dimensional
ART: assisted reproductive technology
CPA: cryoprotectants
FRS: female reproductive system
HUVEC: human umbilical vein endothelial cell
PDMS: polydimethylsiloxane
PMMA: polymethyl methacrylate
