# Supplementary material for: Microfluidic chips in female reproduction: a systematic review of status, advances, and challenges
Source: Theranostics. 2024 Jul 15;14(11):4352–74. doi: 10.7150/thno.97301 (PMC11303079; doi:10.7150/thno.97301)
Supplement: Supplementary file 1 — Supplementary table. [file thnov14p4352s1.pdf]

Table S1. Search terms of databases (from 1/1/2010 to 31/12/2023).

Database	Search term	Number
**PubMed**	((ovary) OR (follicle) OR (oocyte) OR (fallopian tube) OR (oviduct) OR (uterus) OR (endometrium) OR (womb) OR (hystera) OR (vagina) OR (cervix) OR (embryo) OR (placenta) OR (fetal membrane) OR (amnion) OR (decidua)) AND ((microfluidic) OR (chip))	2,750
**Scopus**	(TITLE-ABS-KEY (ovary OR follicle OR oocyte OR fallopiantube OR oviduct OR uterus OR endometrium OR womb OR hystera OR vagina OR cervix OR embryo OR placenta OR fetalmembrane OR amnion OR decidua) AND TITLE-ABS-KEY (microfluidic OR chip))	4,673
**Web of Science**	TS=(ovary or follicle or oocyte or fallopian tube or oviduct or uterus or endometrium or womb or hystera or vagina or cervix or embryo or placenta or fetal membrane or amnion or decidua) AND TS=(microfluidic or chip)	6,546
**ScienceDirect**	Title, abstract, keywords: (ovary OR follicle OR oocyte OR fallopiantube OR oviduct OR uterus OR endometrium OR womb OR hystera OR vagina OR cervix OR embryo OR placenta OR fetal membrane OR amnion OR decidua) AND (microfluidic OR chip)	512
**IEEE Xplore**	(("ovary" OR "follicle" OR "oocyte" OR "fallopian tube" OR "oviduct" OR "uterus" OR "endometrium" OR "womb" OR "hystera" OR "vagina" OR "cervix" OR "embryo" OR "placenta" OR "fetal membrane" OR "amnion" OR "decidua") AND ("microfluidic" OR "chip"))	188
